# A Radiological Review of Ewing's Sarcoma of Mandible: A Case Report with One Year Follow-up

**DOI:** 10.5005/jp-journals-10005-1200

**Published:** 2013-08-26

**Authors:** KB Bimal Krishna, Valsa Thomas, Jayasree Kattoor, P Kusumakumari

**Affiliations:** Postgraduate Student, Department of Oral Medicine and Radiology Government Dental College, Thiruvananthapuram, Kerala, India e-mail: drbimalkrishna@gmail.com; Professor and Head, Department of Oral Medicine and Radiology Government Dental College, Thiruvananthapuram, Kerala, India; Professor and Head, Department of Pathology, Regional Cancer Centre, Thiruvananthapuram, Kerala, India; Professor and Head, Division of Pediatric Oncology, Regional Cancer Centre, Thiruvananthapuram, Kerala, India

**Keywords:** Ewing's sarcoma, Mandible, Malignant

## Abstract

Ewing's sarcoma (ES) is an uncommon round cell tumor with an aggressive course affecting mainly children and young adults. Only 1% of cases is reported with jaw involvement and have mandibular predilection. Radiographic finding in ES reflect many destructive nature of the lesion, like osteolysis, cortical erosion, periostitis and soft tissue mass. A case of ES of the mandible is reported with special consideration to the radiological appearance.

**How to cite this article:** Krishna KBB, Thomas V, Kattoor J, Kusumakumari P. A Radiological Review of Ewing's Sarcoma of Mandible: A Case Report with One Year Follow-up. Int J Clin Pediatr Dent 2013;6(2):109-114.

## INTRODUCTION

Tumors exhibiting neuroectodermal differentiation occur throughout the body, and the diverse tissues of the head and neck gives rise to a wide assortment of these neoplasms. Neuroectodermal neoplasms may be divided into lesions showing primarily epithelial differentiation (group I) and a more diverse group of nonepithelial neoplasms with predominantly neural features (group II) includes Ewing's sarcoma (ES).^1^ Since its original description by James Ewing in 1921, ES remains an enigmatic malignancy. ES is a poorly differentiated tumor of uncertain histogenesis and aggressive biologic behavior, and has emerged as one of most common sarcoma of childhood.^2^ The basic radiographic finding in ES is predominantly permeative osteolysis with a soft tissue mass. ES can mimic most malignant and a few benign entities of bone and referred as ‘great imitator of bone pathology’. This paper reports a case of ES of the mandible in a 3.5 years old girl, highlighting pre- and post-treatment radiological features.

## CASE REPORT

**Fig. 1:**
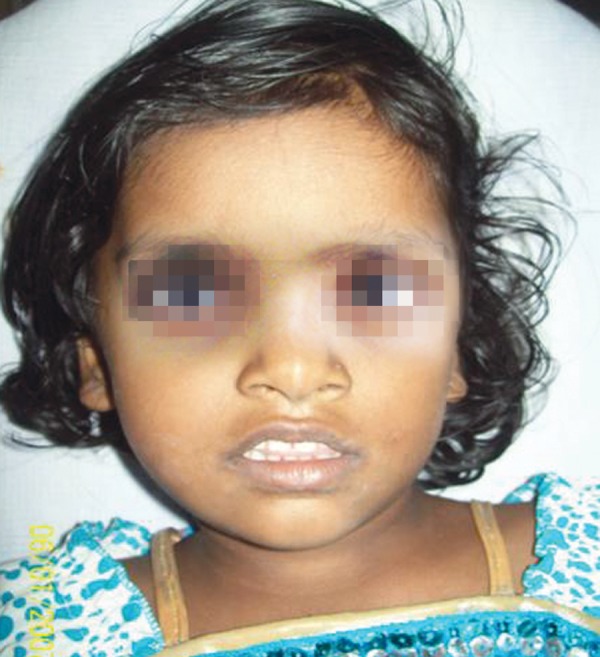
Extraoral clinical photograph showing minimal right side lower facial swelling

A 3.5 years old female child was brought to department of oral medicine and radiology with a complaint of mobility of right lower back tooth of 1 week duration. There was no history of toothache, trauma or associated symptoms. Extraoral examination revealed an irregular lobulated, nontender bony hard swelling of size 3 × 3.5 cm with uniformly blending borders on buccal aspect of right side body and angle of mandible (Fig. 1). Lower border of the mandible showed a discontinuity without considerable expansion. It extends to midline through submandibular region with a size of 4 × 5 cm. Local rise in temperature noted on overlying skin and is not fixed to swelling.

**Fig. 2:**
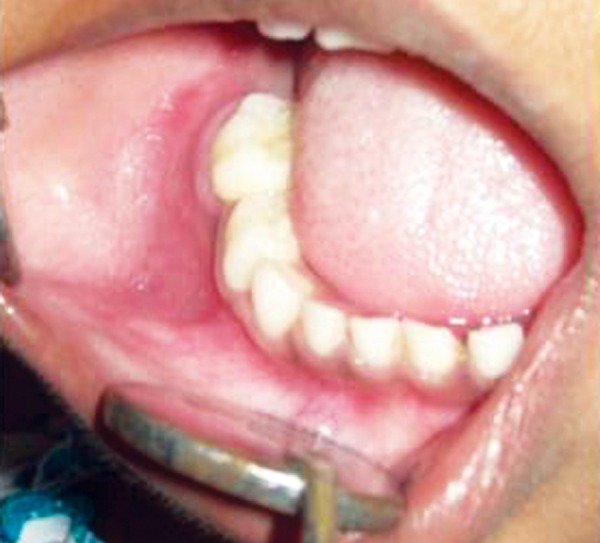
Intraoral clinical photograph showing minimal right side buccal sulcus swelling

Intraorally, right lower gingivobuccal sulcus and floor of mouth obliterated due to swelling from 84 region to retromolar region (Fig. 2). An irregular lobulated swelling of variable consistency with bilateral cortical plate expansion was seen on premolar-molar region. Hard tissue examination showed full complement of healthy teeth except grade III mobile nontender, 85.

**Fig. 3:**
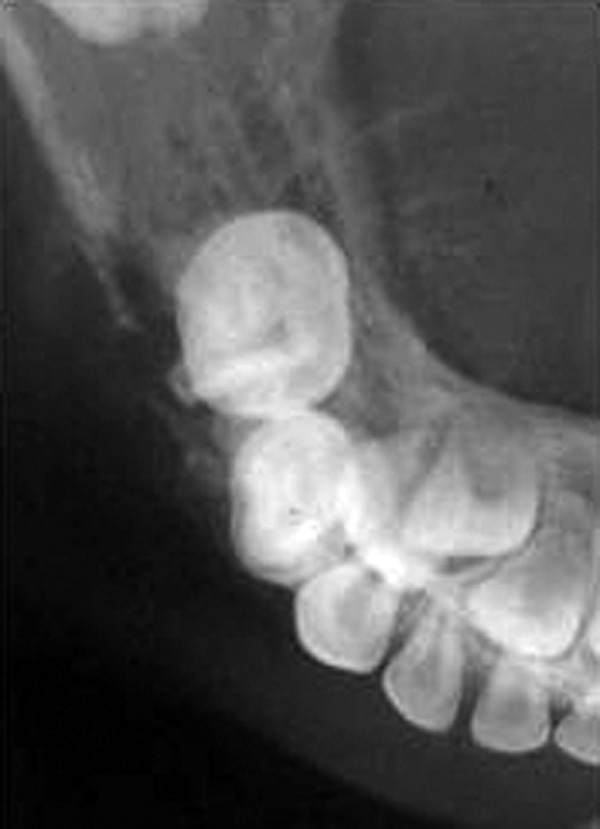
Cropped pretreatment occlusal radiograph shows radiolucent lytic lesion with cortical erosion and bony spiculations

**Fig. 4:**
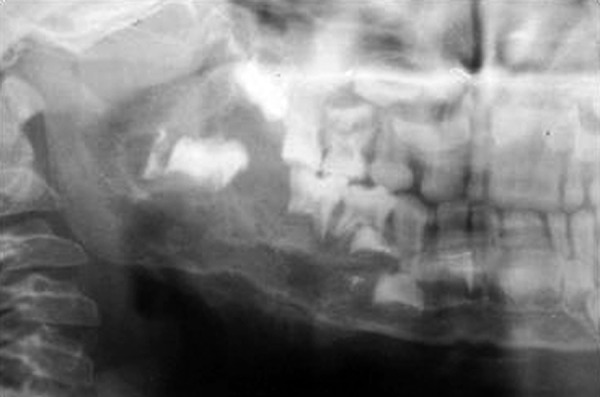
Cropped pretreatment panoramic radiograph shows radiolucent ill-defined lytic lesion with bony spiculations, teeth displacement and missing 45

**Fig. 5:**
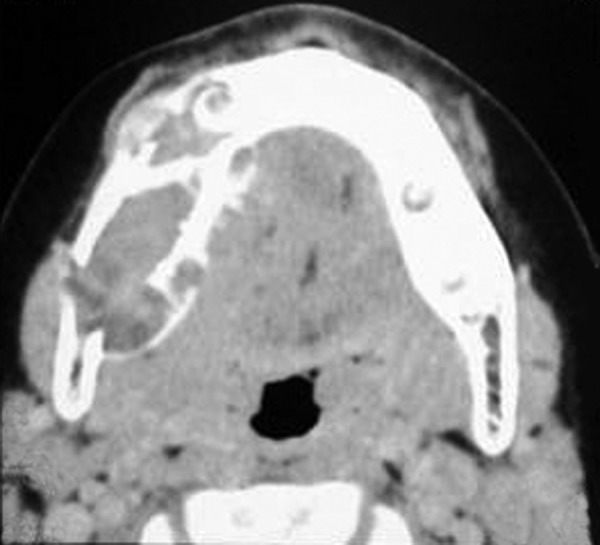
CT axial section shows multilocular expansile lytic lesion in body of mandible right side with significant enhancing soft tissue matrix

Occlusal and panoramic radiographs revealed ill-defined lytic lesions involving premolar-molar region of right side body of mandible, extending from 83 to angle of mandible. Cortex and periosteum was characterized by erosion, thinning and discontinuity on buccal cortex; radiating spicules on lingual cortex; irregular thinning of lower cortical plate seen without considerable expansion. Multiple irregular patchy radiolucent areas in internal structure of permeative and moth eaten pattern with size varying from 0.5 to 2 cm was noted in internal structure (Fig. 3). Considerable root resorption was seen on 84, 85. Other effects on surrounding structures include posterior superior displacement and mesial tipping of developed crown of dental follicles of 46, 47 and missing dental follicle of 45. Loss of alveolar canal outlines medial to angle of mandible (Fig. 4). Computed tomographic (CT) axial section showed multilocular expansile lytic lesion in body of mandible right side with significant enhancing soft tissue matrix (Fig. 5).

**Fig. 6:**
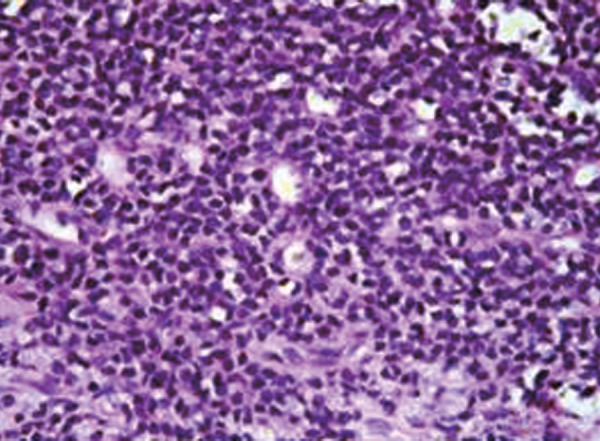
H&E stain shows round cells with scanty cytoplasm and pleomorphic round or oval nuclei

**Fig. 7:**
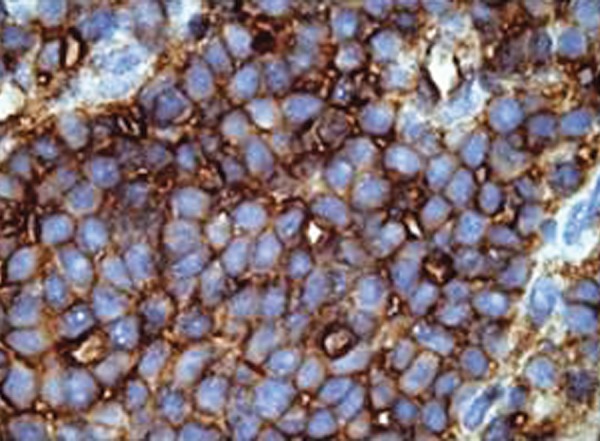
The tumor cells show membranous expression of MIC2

Trucut needle biopsy showed spicules of bone with a cellular cytoplasm composed of round cells with scanty cytoplasm and pleomorphic round or oval nuclei in sheets and sinusoidal pattern (Fig. 6). Immunohistochemistry showed diffuse strong membrane positivity for MIC2 (Fig. 7), focal positivity for synaptophysin and negativity for desmin and diagnosis came compatible with ES or primitive neuroectodermal tumor (PNET).

**Fig. 8:**
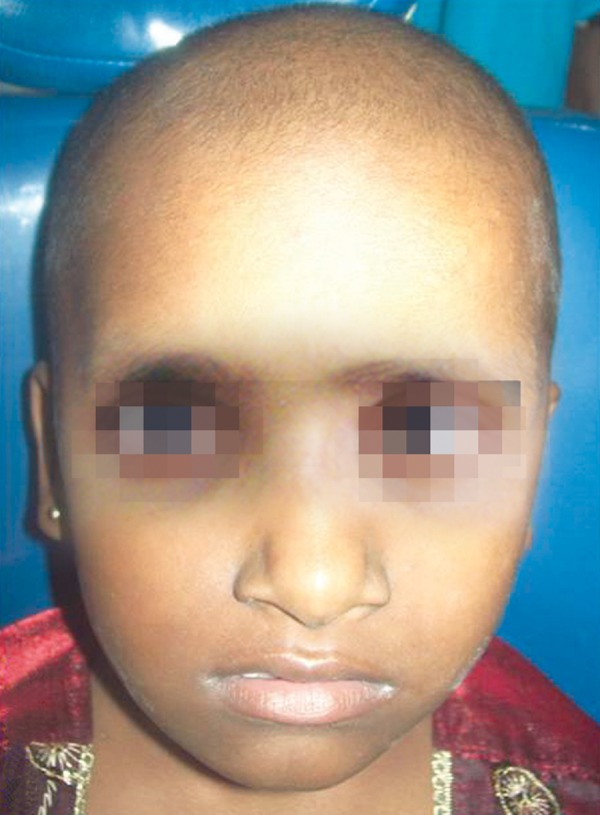
Post-treatment extraoral clinical photograph showing minimal right side lower facial swelling

**Fig. 9:**
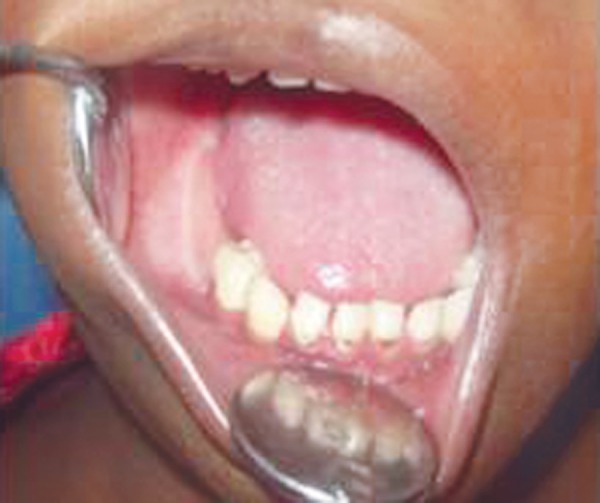
Intraoral clinical photograph with missing 85

Patient underwent radiotherapy and adjuvant chemotherapy treatment with vincristine, cyclophosphamide, etoposide and mesna in Regional Cancer Centre, Thiruvananthapuram, Kerala. One year follow-up clinical review showed bony hard nontender diffuse expansion of right side mandible without apparent soft tissue swelling extraorally (Fig. 8). Intraorally missing 85 was noted due to exfoliation, 6 months after onset of treatment (Fig. 9).

**Fig. 10:**
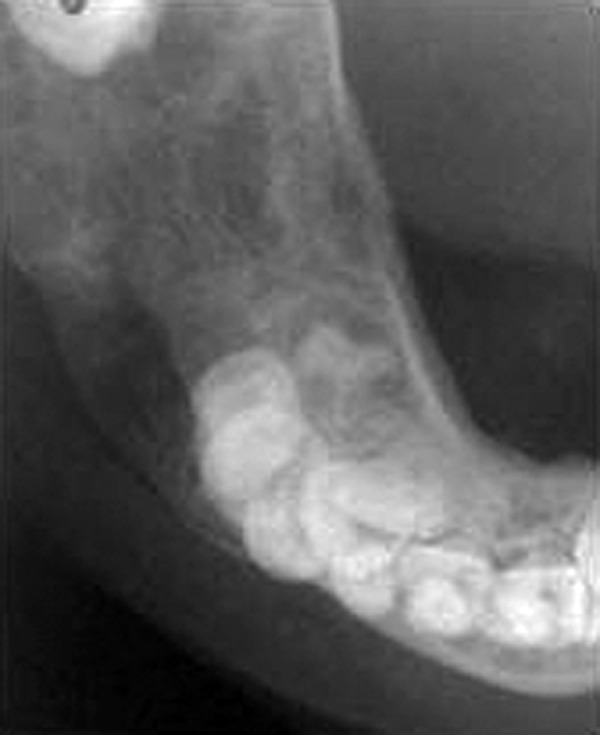
Cropped post-treatment occlusal radiograph shows intact cortex and mixed radiodensity in internal structure

**Fig. 11:**
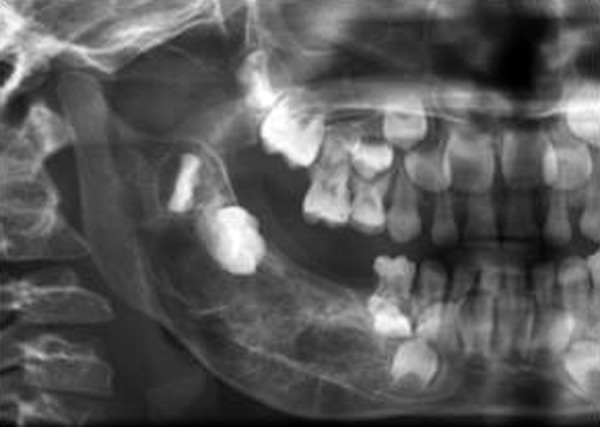
Cropped post-treatment panoramic radiograph shows uniform expansion with cortical regeneration throughout the lower border of the mandible. Mixed radiodensity seen in internal structure

Radiological follow-up using occlusal and panoramic radiography after 1 year of treatment showed mixed radiodensity on premolar-molar region and anterior border of ramus of right side of mandible involving 82 to 47. Intact and visible uniform expansion with cortical regeneration was seen throughout the lower border of the mandible near the lesion. Cortical break in buccal aspect remodeled with overzealous cancellous bone apposition resulted in an increased buccolingual width. Periosteal reactions including vertical spiculations completely resolved and cortical out line re-established in lingual aspect (Fig. 10). Considerable reduction in permeative and moth eaten pattern and replacement with accentuated multiple linear and granular trabeculae with a multiseptated pattern was noted in internal structure. Enamel formation completed and dentine formation started 44 and 47. Relative developmental delay was observed on 46 (Fig. 11). Radiological features suggestive of cessation of malignant tissue growth and active destruction, followed by vigorous regeneration and reparative reactions of healthy osseous and dental tissues and arrested growth of dental follicle near epicenter of the lesion.

## DISCUSSION

ES first described by James Ewing who was a pioneer in the field of cancer research, in 1921. ES is related to the PNET, sharing a common karyotype translocation t(11;12)(q24;q12) in approximately 90% of these tumors. This translocation results in juxtaposition of the ENS and the FLI1 genes. The current research found that both ES and PNET showed similar translocations and is considered to be the ends of a histological spectrum of ‘Ewing's family of tumors’ (EFT).^3^

ES accounts for approximately 6% of all malignant bone tumors. Approximately 4% of ES have arisen in the bones of the head and neck, with 1% occurring in the jaws. When the jaws are involved, the predilection is for the ramus of the mandible, with few cases reported in the maxilla. Posterior parts favored over anterior parts. Because ES has a propensity to metastasize to other bones, the possibility that jawbone involvement represents metastatic disease from another skeletal site should always be considered.^4,7^ ES has a predilection for the male sex (male:female ratio, 1.3-1.5:1). Peak incidence is during the second decade of life; although 20 to 30% of cases are diagnosed in the first decade. The age of the patient is important diagnostically. When confronted with patients older than 30 years, the clinician must first eliminate other small round-cell tumors, including small-cell carcinoma and large-cell lymphoma, before making a diagnosis of ES. In patients younger than 5 years, the possibility of metastatic neuroblastoma or acute leukemia needs to be ruled out.^5^ An extensive review of the literature shows only few cases of ES affecting children under 5 years of age.^6^ In the case, a 3.5 years old female child represented an uncommon case of ES with lower extreme of age at the time of diagnosis.

The common presenting signs and symptoms in maxillofacial region includes swelling, pain, loose teeth, paresthesia, ulceration, trismus and toothache. Extraoral features reported are exophthalmos, ptosis, epistaxis, otitis media and sinusitis.^7^ Other systemic symptoms and signs include fever, anemia and nonspecific signs of inflammation, such as increases in ESR, moderate leukocytosis and an increase in serum LDH. Conventional blood, serum and urine tests cannot specifically identify ES.^5^ In this case, tooth mobility was the first symptom with a progressive painless swelling. Patient was afebrile and hematological findings showed a lymphocyte predominant smear and a mild eosinophilia.

The basic radiographic finding in ES reflect the aggressive nature of the lesion, including osteolysis, cortical erosion, periostitis and soft tissue mass.^5^ According to Hofer et al lytic malignant diseases in the jaws manifests mainly as moth eaten and permeative patterns. ES can mimic most malignant and a few benign entities of bone, such as osteomyelitis, eosinophilic granuloma, giant cell tumor, simple bone cyst, aneurysmal bone cyst. McCormack et al referred ES as the ‘great imitator of bone pathology’ due to its clinical presentations.^8^

Features of ES of mandible reported by various authors in articles from year 2000 searched manually and electronically using PubMed are depicted in Table. 1.^9-16^

In our case, radiological features noted include multiple ill-defined lytic medullary lesions, cortical erosion and disruption without considerable expansion, effects on adjacent anatomical structures, periosteal reactions and an associated soft tissue mass. Tumor-related osteolysis and periosteal reactions suggest a diagnosis of primary malignant tumor.

Permeative destruction is a major feature of ES seen in approximately 90% of cases and ranges from pin head sized holes, moth eaten, rotten wood, geographic or nearly purely lytic pattern with or without associated sunray spicules of the periosteal bone. Ill-defined lytic patterns seen in this case include moth eaten and permeative pattern which represent poorly defined areas of medullary destruction. Buccal cortical erosion extending from medullary destruction and disruption without considerable expansion in this case are seen almost exclusively in rapidly destructive malignant disease and in some cases of metastatic disease. Destructive osteolytic lesion of ES should be differentiated radiologically from osteosarcoma, neuroblastoma, lymphosarcoma, histiocytosis-X, osteomyelitis, rhabdo-myosarcoma and metastatic carcinoma.

Wood et al in a review of 105 cases reported laminar periosteal response like onion-peel appearance was not a common feature of ES in jaw bones.^7^ Periosteal reactions are the reactive osteogenesis of the periosteum, are caused by extraosseous extension of the tumor. Several types of periosteal reactions have been observed in ES: (i) an ‘onion-peel appearance’ is a multilayered reaction, (ii) a ‘sunburst’ or ‘spiculae’ pattern is a perpendicular reaction, while (iii) ‘Codman's triangle’ is a triangular lifting of the periosteum from the bone at the site of detachment. Sun-ray like vertical spiculations and supraperiosteal soft tissue mass were noted in this case.^7,10^

Radiological findings of ES on adjacent anatomical structures include displacement or destruction of unerupted tooth follicles, advanced eruption, loss of lamina dura, widespread periodontal ligament space. Root resorption, too much eruption for amount of root formation, widened canal, osteosclerosis, antral clouding, etc. were also noted in literature.^7,17^ Current case revealed missing permanent tooth germ, displacement, root resorption of deciduous teeth and absence of inferior alveolar canal outline of premolar-molar region.

Presence of soft tissue mass, age of the patient, hematological investigations and radiology can facilitate differential diagnosis, histopathology and immuno-histochemistry enables final diagnosis.

**Table 1:** **Table 1**: Clinical, radiological and treatment: Summary of ES of mandible reported in literature

*No.*	*Author*	*Age/ sex*	*Location*	*Clinical features*	*Radiological features*	*Treatment and review details*
1.	Luis Gorospe et al^9^	12/F	Ramus of mandible right side	Tender swollen mass	Permeative poorly demarcated destructive lesion with cortical erosion	Chemotherapy, regional hyperfractionated radiotherapy patient is alive and symptom-free, with no evidence of local recurrence or distant metastases
2.	Mubeen etal^10^	18/M	Ramus and condyle right side	Swelling with tenderness, right submandibular lymphadenitis	III-defined radiolucent lesion with destruction and periosteal reaction, with enlargement of muscles of mastication	Undergoing chemotherapy. Patient feeling well
3.	Sergio LPC Lopes etal^11^	14/M	Ramus and angle of right side mandible	Nontender hard immobile lesion with lymphadenopathy	Osteolytic radiolucency, cortical destruction and thinning, periosteal reaction with sunray appearance, Codman's triangle, soft tissue mass	Chemotherapy and radiotherapy. Reduction in tumor volume and patient feeling well
4.	Kourosh Taberi et al^12^	17/F	Ramus and condyle of mandible left side	Tender swelling with inferior alveolar nerve paresthesia	Expansile radiolucent lesion with cortical destruction and soft tissue mass	Surgery, radiotherapy and chemotherapy. No recurrence after 5 years
5.	Marco Tullio etal^6^	4/F	Body of mandible right side	Nontender hard mass with teeth/follicle displacement	Mixed lesion with III-defined borders, displacement of dental follicles, marrow destroying mass with cortical destruction	Treatment with multiagent chemotherapy after the first chemotherapeutic cycle infection developed and resulted in death
6.	Sharada Petal^13^	15/F	Ramus angle and body of mandible right side	Swelling and mobility of teeth	III-defined osteolytic lesion with floating teeth appearance	Patient was treated with neoadjuvant chemotherapy followed by surgical excision and reconstruction
7.	Adriano Santana^14^	35/F	Angle and body of the mandible as well as the left submandibular region and the floor of the oral cavity	Swelling on the leftside of the mandible	Expansive lesion with opacity, compatible with soft tissues, capsulated and with septa, involving the left portion of the mandible and extending to the surrounding tissues	Left hemimandibulectomy, tracheostomy and radiotherapy. The patient has been in follow-up for 4 years and is free of disease and feeling well.
8.	Martin Gosau etal^15^	24/M	Body of mandible right side	Swelling and pain with teeth mobility, hyperesthesia	III-defined diffuse radiolucency	Radical tumor surgery with subtotal mandibulectomy and cervical lymph node dissection. Reconstruction followed by chemotherapy
9.	JP Singh etal^16^	20/F	Ramus of mandible right side	Swelling of the mandible on right side. There was no pain or fever and the swelling was not tender	Lytic permeative destruction of the right ramus of the mandible with soft tissue swelling	The patient was given radiotherapy followed by chemotherapy.

EFT cells show membranous expression of CD99 or MIC2 on immunohistochemistry. Antibody against FLI1, which is centered in the nucleus of the tumor cells, has been shown to be specific for EFT. Depending on the degree of neuroectodermal differentiation, the tumor cells may also express neuron-specific enolase (NSE), synaptophysin and S-100 protein. Immunohistochemistry is essential as the family of small round cell tumors is rather large and includes non-Hodgkin lymphoma, neuroblastoma, rhabdo-myosarcoma, mesenchymal chondrosarcoma, retino-blastoma (Rb), and desmoplastic small round cell tumor (DSRCT). Other tumors can also show small round cells and include osteosarcoma, synovial sarcoma, malignant peripheral nerve sheath tumor and melanoma. Although CD99 shows strong membrane positivity in EFT, it can also be positive in other tumors like lymphoblastic lymphoma, rhabdomyosarcoma, synovial sarcoma, mesenchymal chondrosarcoma, Wilms tumor and rarely in DSRCT. Hence, a panel of immunohistochemical stains is employed to arrive at a definitive diagnosis.^18^

Treatment of ES has undergone significant changes by utilization of integrated therapies, including chemotherapy, radiation therapy and surgery, has led to an impressive improvement in prognosis. Poor prognostic factors are patients below 10 years of age, pelvic lesions, presence of metastasis, presence of systemic symptoms, large tumor volume, high mitotic rate, filigree pattern in histopathological sections and poor response to chemotherapy. ES of the mandible has got better prognosis than long bones since facial sites are diagnosed earlier.^15^ Current case was treated with curative chemotherapy and review of the patient after 1 year did not show any abnormal findings.

A case of ES in a young child highlighting diagnostic radiological features in jaws and after 1 year post-treatment follow-up was reported. Familiarity with radiological findings will enable the clinician to rule out more common inflammatory lesions and narrow down differential diagnosis. Early diagnosis and judicious management ensures good prognosis for this enigmatic malignancy.
